# Kidney protective effects of melatonin

**Published:** 2014-01-01

**Authors:** Maryam Tavakoli

**Affiliations:** Department of Internal Medicine, Isfahan University of Medical Sciences, Isfahan, Iran

**Keywords:** Nephropathy, Melatonin, Renoprotection

**Implication for health policy/practice/research/medical education:**

Recent studies shown that melatonin administration, attenuated oxidative stress, inflammation, and restored renal function and structure in rats. Melatonin could be an attractive adjunctive therapy, since it is a natural, inexpensive, widely available, orally administered and relatively safe product.

Melatonin is a hormone produced in the pineal gland. Melatonin is produced of tryptophan and serotonin and is metabolized to 6-hydroxyl melatonin in the liver (1-3). Melatonin is a highly important antioxidant. Free radicals damages cells. Melatonin is an efficient neutralizer of free radicals (1,2). Melatonin plays an important role in various physiological processes including the regulation of circadian and endocrine rhythms, aging, the stimulation of immune functions and the prevention of the adverse effects of antibiotics, including renal failure (2-5). Melatonin reduces the oxidative induced brain, heart, kidney, and liver damage in rats. These effects of melatonin are related to scavenging of a variety of toxic oxygen and nitrogen based reactants and stimulation of antioxidative enzymes (1-5). Liver and kidney are metabolically highly active in xenobiotic metabolism and excretion, they have, compared to other organs, a greater load of free radical activity and thus are more prone to oxidative damage. Nephrotoxicity is an important side effect of contrast media, aminoglycosides, chemotherapy (2-6). In vivo and in vitro melatonin has been found to protect tissues against oxidative damage generated by a variety of toxic agents and metabolic processes, including chemotherapy induced toxicity and ischemia reperfusion injury in kidney, liver and brain (4-7). Melatonin has recently been found to protect against Adriamycin induced nephrotoxicity, aminoglycosides induced nephrotoxicity, and contrast media induced nephrotoxicity. Studies indicated that pretreatment with melatonin improves dramatically the histological and functional damage in this experimental model (3-7). In summary the studies showed that melatonin administration attenuated oxidative stress, inflammation, and kidney function and structure in rats. If proven effective, melatonin would be an attractive adjunctive therapy, since it is a natural, inexpensive, widely available, orally administered and relatively safe product (1-7).

## Conclusion

Recent studies shown that melatonin administration, attenuated oxidative stress, inflammation, and restored renal function and structure in rats. Melatonin could be an attractive adjunctive therapy, since it is a natural, inexpensive, widely available, orally administered and relatively safe product.

## Author’s contribution

MT is the single author of the paper.

## Conflict of interests

The author declared no competing interests.

## Ethical considerations

Ethical issues (including plagiarism, data fabrication, double publication) have been completely observed by the author.

## Funding/Support

None.
